# The Rise of Staphylococcal Super Antigens in Psoriatic Patients: A Case-Control Study

**DOI:** 10.5812/jjm.9912

**Published:** 2014-05-01

**Authors:** Najmolsadat Atefi, Samileh Noorbakhsh, Sahar Ghavidel Darestani, Azardokht Tabatabaei, Mohammadreza Rezaee

**Affiliations:** 1 Research Center of Pediatric Infectious Diseases, Iran University of Medical Sciences, Tehran, IR Iran; 2 Head and Neck Surgery Research Center, Tehran University of Medical Sciences, Tehran, IR Iran; 3 Department of Dermatology, Iran University of Medical Sciences, Tehran, IR Iran

**Keywords:** Psoriasis, Enterotoxin F, Staphylococcal, Staphylococcal Enterotoxin (A, B, D)

## Abstract

**Background:**

*Staphylococcus aureus*, the major virulence factor of hospital and community acquired infections, secretes numerous exotoxins (super antigens), which may affect immunological and inflammatory status in psoriatic skin lesion.

**Objectives:**

This study is designed to compare the *S. aureus* super antigens level in sera of psoriatic patients with normal cases (nevus).

**Patients and Methods:**

A case control study was performed in dermatology ward of Rasoul Hospital in Tehran, IR Iran (2008 - 2010). Staphylococcal super antigens (Entrotoxin A, B, D and TSST1) were measured in serum of 41 psoriatic patients and 28 normal persons (Nevus) by ELISA. Chi square values (CI 95%, P value < 0.05) were calculated for all categorical variables.

**Results:**

In this study 63.4% (26) of cases were male, 36.6% (15) were female. Age ranged from 4 months to 64 years old, with a mean age of 33.7 ± 15.4 years. Type of skin disease in cases: 20% (8) were inflicted by the Gutate form of the disease; 59% (23) with chronic plaque psoriasis (CPP), 7.7% (3) with erythrodermic and 12.8% (5) had other types of the disease (plaque, pustular, inverse). TSST (toxic shock syndrome toxin) was detected in 47% (20/41) of cases and in 6% (1/28) of the controls with a significant difference. (P value = 0.000) Entrotoxins (A, B, D) were detected in the sera of 48.8% (21/41) of cases; and only 6 %( 1/21) of controls, showed significant differences (P value = 0.000) positive TSST was more common in spring, and correlates with CPP type of psoriasis, but not related to patient`s gender and age.

**Conclusions:**

In this study, *S. aureus* were 25 times more in psoriatic patients. Super antigens should be first detected in the serum samples; if negative, the skin lesions should be examined by PCR especially in chronic types of disease. Adding the antibiotics against *S. aureus* to other conventional treatments might be helpful. It has a more important and significant role in children with acuteinfection.

## 1. Background

Psoriasis is a skin disease with unknown genetic and immunological pathogenesis. However, activation of T-cells is considered as an important factor in the pathogenesis of this disease, since the laboratory studies have shown that the population of T-cells isolated from the skin of patients with psoriasis is capable to stimulate keratinocytes proliferation (1). Super antigens, including a group of viral or bacterial proteins, can directly bind to major histocompatibility complex (MHC) class II and the Vβ component of T-cell receptors, and cause T-cells activation (2, 3). Recently, much attention has been paid to the role of super antigens as triggering factors in the pathogenesis of psoriasis. Super antigens include a group of bacterial products that are presented to T-cell Receptors (TCR), particularly the Vβ area, after binding to molecules of MHC class II. These molecules are significantly different from common peptide antigens (4).

Super antigens produced by (4) *Staphylococcus aureus* are among the most lethal toxins. Toxins of this large family trigger an excessive cellular immune response leading to toxic shock (5). Some examples of staphylococcal super antigens are staphylococcus enterotoxin A, B, and C (SEA, SEB, SEC), toxic shock syndrome toxin-1 (TSST-I) and exfoliative toxin (ET). Staphylococcal super antigens (SAg's) play role in the pathogenesis of inflammatory skin diseases. Severity of PS is significantly correlated to enterotoxin production of the isolated *S. aureus* strains (6). There are different methods to measure the toxin including immunediffusion, ELISA and agglutination, but amplification methods such as PCR that detect responsible genes for TSST-I, SEC, SEB are highly sensitive and specific. In various studies, the evidence showed their cellular effects on the pathogenesis of psoriasis (4, 7).

Detection of new methicillin-resistant *S. aureus* isolates containing the toxic shock syndrome toxin 1 gene that is responsible for hospital- and community-acquired infections in France was reported by Durand et al. (8). Like other countries, methicillin resistant *S. aureus* in Iran is a major problem (9-12).

## 2. Objectives

In the present study, the role of staphylococcus super antigens in patients with psoriasis was assessed with identification of TSST-I, SEB, SEC in sera (EIA) of cases and normal controls (nevus).

## 3. Patients and Methods

This case control study was done in the dermatology ward of Rasoul Hospital in Tehran, IR Iran during 2008-2010. This study was approved by the Ethics Committee of Research Center of Pediatric Infectious Diseases affiliated to Iran University of Medical Sciences, and performed according to the Declaration of Helsinki Principles. Our study group consisted of 40 patients (26 males and 14 females) with confirmed psoriasis selected continuously by dermatologist by simple sampling method. The control group consisted of 28 non-psoriatic individuals who were referred for cutaneous nevus removal.

A specialist intern visited all the participants for other disorders (immune deficiency states; diabetes mellitus, renal failure; etc.) before enrollment. All of the participants were fully informed about the study and they signed the informed consent forms before enrollment. Inclusion criterion: clinical diagnosis of plaque type psoriasis that was pathologically confirmed. Exclusion criteria: patients with immune deficiency states; diabetes mellitus, renal failure; a history of treatment with oral or topical antibiotics or PUVA therapy in the past two weeks were excluded. 

### 3.1. Data Collection

Initially, a questionnaire was completed by an authorized physician for each case and control.

Blood samples (2 mL) were taken, centrifuged and transferred to research laboratory. The sera were restored at -20˚C until the serologic examination was performed. Serum level of staphylococal super antigen sentrotoxins A, B, C, TSST1 were measured by EIA methods with commercial kits (ABCam Inc, UK). The results were interpreted as suggested by the manufacture's instruction. Results were calculated quantitatively. The level of super antigens was compared between cases and controls.

### 3.2. Statistics Analysis

All analyses were conducted using SPSS; version 11.5. The Student’s t-test was used to determine significant differences in means for all continuous variables. Chi-square values (CI 95%, P < 0.05) were calculated for all categories. P < 0.05 was considered significant. The likelihood ratio measured to test the linear trend and interaction between variables. McNamara and computing kappa statistics were used to compare variables.

## 4. Results

In this study, 63.4% (26) of cases were male and 36.6% (15) were female. The age ranged from 4 months to 64 years with a mean of 33.7 ±15.4 years. Type of skin disease: 20% (8) were inflicted by the Gutate form of the disease; 59% (23) with CPP, 7.7% (3) with erythrodermic and 12.8% (5) had other types of the disease (plaque, pustular, inverse). TSST was detected in 47% (20/41) of cases and 6% (1/28) of controls with a significant difference (P value = 0.000).

Entrotoxins (A, B, D) were detected in sera of 48.8% (21/41) of cases and 6% (1/21) of controls with a significant difference (P value = 0.000). Positive test had not related to gender (P value > 0.05). Cases with positive TSST were more common in spring, but this preference was not seen in other toxins (P value = 0.25). Positive TSST correlates with CPP type of psoriasis. The mean age of cases with positive and negative toxins showed no differences.

## 5. Discussion

Here we observed the highest rate of positive results for all types of staphylococcal toxins (super antigen) in psoriatic cases. Positive TSST was obtained in 47% of cases *vs.* 6% of controls (P value = 0.000); also positive enterotoxins (A, B, D) were detected in 48.8% of cases *vs. *6% of controls (P value = 0.000), no differences were detected in patients in different age group. In contrast to other toxins, positive TSST was more frequent in spring and in cases with CPP type of the disease. Some other studies, like ours, have shown that bacterial toxins might play a major role in the process of induction or exacerbation of psoriasis lesions. Balci et al. (13) reported the high prevalence of *S. aureus* cultivation and super antigen production in patients with psoriasis.

Tomi et al. (4) isolated *S. aureus* from 60% of their psoriasis patients out of whom 36% were toxin producers, and 12% of healthy controls who all lacked *S. aureus* toxin. In another study, El Ferezli et al. (14) explained the clinical and therapeutic implications of patients with psoriasis who possess genes that encode the toxins (superantigens) of *Streptococcus* sp. and *S. aureus* isolates.

Floret et al. discussed clinical aspects of streptococcal and staphylococcal toxin related diseases. Seven super antigens produced by *S. aureus* are amongst the most lethal toxins which trigger an excessive cellular immune response leading to toxic shock (15). In another study by Kaempfer et al. (16) the antagonist activity of this peptide was reported, thus they identified a novel domain in super antigens that is critical for their toxic action. A recent study in our center (PCR methods) had shown similar results. This study showed isolation of toxin producing *S. aureus* in 6.5% of plaque type psoriasis patients and 2.5% of the controls. The significant difference suggests the possible role of bacterial super antigens in the pathogenesis of psoriasis (12).

In this study, the level of *S. aureus* super antigens is 25 times more in psoriatic patients. Super antigens should be first detected in sera; if negative, the skin lesions should be examined by PCR especially in chronic types of the disease. Adding antibiotics against *S. aureus* to other conventional treatments might be helpful. It has an even more important and significant role in children with chronic infections.
